# Food Consumption Patterns and Body Composition in Children: Moderating Effects of Prop Taster Status

**DOI:** 10.3390/nu11092037

**Published:** 2019-08-31

**Authors:** Lee Stoner, Nicholas Castro, Anna Kucharska-Newton, Abbie E. Smith-Ryan, Sally Lark, Michelle A. Williams, James Faulkner, Paula Skidmore

**Affiliations:** 1 Department of Exercise and Sport Science, University of North Carolina at Chapel Hill, Chapel Hill, NC 27599, USA; 2 School of Sport & Exercise, Massey University, Wellington 6021, New Zealand; 3 School of Kinesiology and Nutrition, University of Southern Mississippi, Hattiesburg, MS 39406, USA; 4 Department of Epidemiology, The Gillings School of Global Public Health, University of North Carolina, Chapel Hill, NC 27599, USA; 5 Department Epidemiology, Harvard T.H. Chan School of Public Health, Boston, MS 02115, USA; 6 University of Sport & Exercise, University of Winchester, Winchester S022 4NR, UK; 7 Department Medicine, University of Otago, Christchurch 8011, New Zealand

**Keywords:** children, obesity, overweight, bitterness, breakfast, processed foods, fruits and vegetables

## Abstract

This cross-sectional study determined whether 6-n-propylthiouracil (PROP) taster status moderates the relationship between food consumption patterns and body composition in children. Children were recruited (*n* = 342, 50% female, 8–10 y) from across New Zealand. Using a food frequency questionnaire, these food consumption patterns were derived: Processed Foods, Fruit and Vegetables, and Breakfast Foods. Body composition variables included: body fat (%), fat mass (kg), fat mass index (FMI, kg/m^2^), body mass index (kg/m^2^) and waist to height ratio (W:Ht). Following adjustment for confounders, Processed Foods were positively associated with %fat (*p* = 0.015), fat mass (*p* = 0.004) and FMI (*p* = 0.016). Taste test strips determined PROP status. For Breakfast Foods, there were small negative associations with all body composition variables (*p* ≤ 0.001 to 0.037). The population sample was also stratified by PROP taster status. For the non-tasters, there were small to moderate negative associations between Breakfast Foods and each body composition variable (*p* = 0.003–0.045) except W:Ht (*p* = 0.112), and these relationships were stronger for girls compared to boys. For the tasters, there were small to moderate positive associations between Processed Foods with %fat (*p* = 0.030), fat mass (*p* ≤ 0.001) and FMI (*p* = 0.014). In conclusion, sensitivity to bitterness may moderate the relationship between food consumption patterns and body composition in children.

## 1. Introduction

Similar to the global trend, the prevalence of obesity in New Zealand continues to rise, with a recent report from the Organization for Economic Cooperation and Development (OECD) ranking New Zealand as the third worst country for obesity [1]. More concerning are the high obesity rates among New Zealand children (aged 0–14 years), with 20% overweight and a further 12.4% obese in 2017/2018 [2]. Contributory factors to this obesity ‘epidemic’ include decreased levels of physical activity and an increase in adverse eating behaviors [3], including the consumption of energy-dense high-fat foods and low fruit and vegetable consumption [4]. Food preference has previously been shown to be impacted by individual variation in human olfactory and taste receptors [5].

While humans can experience a vast arrange of food flavors, these flavors are triggered by only a few distinct taste modalities: sweet, salty, sour, umami (savory), and bitter [6,7]. Bitterness, which has also been associated with taste acuity, is the most widely studied taste modality [8,9,10]. Sensitivity to bitterness can be assessed by determining sensitivity to 6-n-propylthiouracil (PROP), a bitter thiourea compound which elicits a bitter taste stimulus in some individuals. It has been hypothesized that individuals with increased bitter taste sensitivity might avoid antioxidant-rich vegetables because of their perceived bitterness, instead consuming sweet and fatty foods. However, an increased sensitivity to bitterness has also been associated with heightened taste acuity [8,9,10], which may prevent food overconsumption. This might explain why findings in children are mixed, with several studies reporting associations between PROP status and liking and/or intake of fat-containing foods [11,12,13], and other studies reporting no relationship between PROP status and intake of high-fat foods [14,15]. Similarly, a number of studies in children have reported higher intake of bitter tasting vegetables for non-tasters [16,17,18], whereas other studies have reported no differences as a function of PROP status [11,13,14]. According to a review by Keller et al. [12], some inconsistencies across studies might be explained by small sample size and the methodology used to capture diet. Specifically, Keller et al. [12] argue that the majority of studies that have found differences between dietary intake as a function of PROP status have measured ad libitum food intake or utilized food frequency questionnaires (FFQs), whereas most studies that have used self-report diet recalls or food diaries have not seen any relationship with PROP status. A plausible explanation is that although food diaries and 24-h dietary recalls are considered more accurate for assessing total energy intake, they may not capture sufficient variability in the foods or patterns of foods.

Studies investigating associations between PROP status and body composition in children have also produced mixed findings. Several studies have reported an association between PROP status and BMI, with some reporting higher BMIs for tasters [15,19], others reporting higher BMIs for non-tasters [13,20,21], and some reporting no relationship [12,14,22]. It is likely that the relationship between PROP status and body composition is not categorical. That is, it is likely that PROP status does influence food choice, and in turn food choice may influence metabolism and overweight/obesity [10]. However, we are not aware of any large studies that have measured food consumption patterns in children using an FFQ, and which have explored whether PROP taster status moderates the relationships between food consumption patterns and overweight/obesity. 

The purpose of the current study was to explore whether PROP taster status moderates the relationship between food consumption patterns and body composition in pre-adolescent children. This relationship was examined in a representative sample of children aged between 8 and 10 years of age in New Zealand. Food consumption patterns were determined using a validated FFQ [23], as this method may be superior at capturing food consumption patterns compared to other methodologies. Statistical models were adjusted for confounders which may influence food choice or body composition, including socioeconomic status (SES), ethnicity, age and sex. Additionally, models were adjusted for weekly physical activity levels, as physical activity may influence energy intake and expenditure.

## 2. Materials and Methods 

This observational cross-sectional study is reported in accordance with the Strengthening the Reporting of Observational Studies in Epidemiology (STROBE) guidelines [24]. This paper is part of a larger cross-sectional study: Pre-Adolescent Cardio-Metabolic Associations and Correlates, (PACMAC, ACTRN12614000433606) [25]. Parental/guardian consent and child assent were obtained prior to participation, in accordance with the requirements of the New Zealand Health and Disability Ethics Committees (HDEC:14/CEN/83). 

### 2.1. Participants

**Figure 1:**
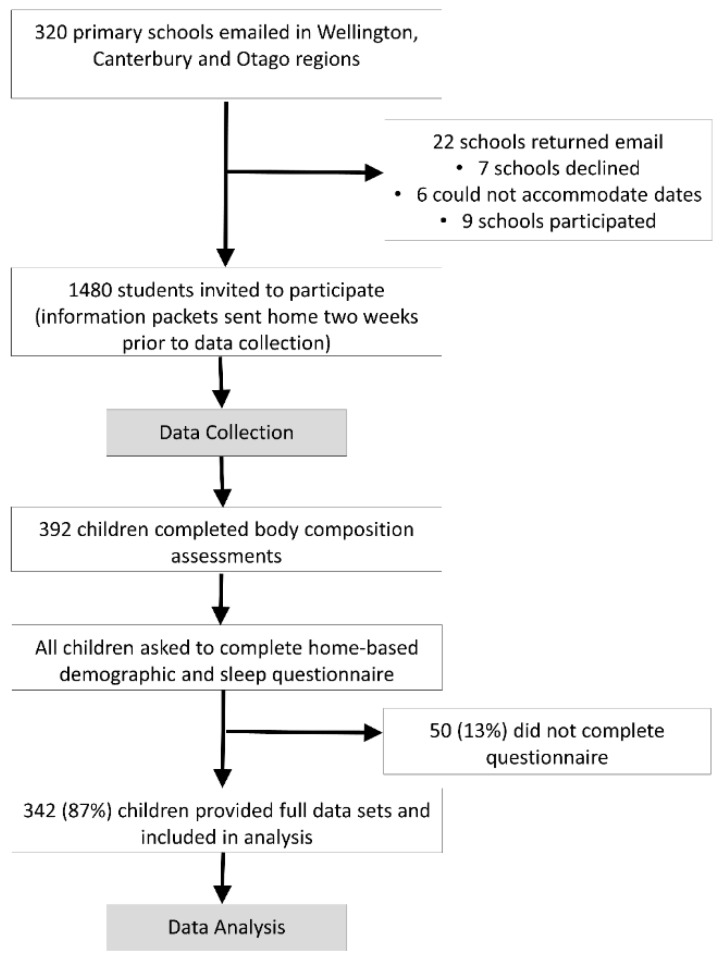
Participant recruitment flowchart.

Children aged between 8 and 10 years of age were recruited from schools in three major cities in New Zealand (Wellington, Christchurch, and Dunedin), between April 2015 and April 2016 (Figure 1). In New Zealand, nearly all schools are publicly funded and currently classified by the predominant socioeconomic status (SES) of attending students in a decile classification system. Within each city, we classified schools as low SES (Deciles 1 to 5) or high SES (Deciles 6 to 10), and randomly sampled from within these strata to approach for participation. Within schools all children in the appropriate age range were eligible for participation, except those prescribed any cardiovascular medications, or with an orthopedic injury in the past three months.

### 2.2. Study Design

All anthropometric and body composition measurements were made in the participants’ schools, on a single day, between 09:00 and 12:00 h. Children were asked to have been fasting for at least three hours, having consumed only water, and to have refrained from exercise for 24 h before assessment. Within 7 days of body composition assessments, dietary patterns, demographics and physical activity levels were collected using a questionnaire (see Supplement Materials). The questionnaires were completed at home jointly by the primary caregiver and child using an online survey (Lime Survey, open source) where available. If this was not possible, a paper copy was provided.

### 2.3. Demographic Data

Collected demographic data included date of birth, sex, self-reported ethnicity, and school address. The school address was used to determine school decile rating, a SES indicator of the school (see above). 

### 2.4. Dependent Variables: Body Composition

Five dependent variables were recorded: body fat (%), fat mass (kg), fat mass index (FMI, kg/m^2^), waist to height ratio (W:Ht), and body mass index (BMI, kg/m^2^). 

#### 2.4.1. Body Fat and Fat Mass

Body fat (%) and fat mass (kg) were measured using multi-frequency bio-impedance analysis (BodyStat Quadscan 4000, Isle of Man, UK), which has previously been validated against deuterium oxide dilution in children [26]. Following at least 5 min supine rest, participants were studied in the supine position on a non-conductive surface, with arms and legs abducted at a 30–45° angle from the trunk to avoid medial body contact by upper and lower extremities. The instrument was calibrated in accordance with the manufacturer’s instructions, and measurements were conducted according to standardized procedures [27]. Two electrodes were placed on the right foot, under 4th/5th toes and proximal end of foot, and two electrodes on the right hand, under 4th/5th fingers and below head of ulna. FMI was calculated by dividing fat mass (kg) by height squared (m^2^). 

#### 2.4.2. Anthropometrics

To calculate the anthropometric indices (BMI and W:Ht), height was measured to the nearest 0.1 cm with a calibrated portable stadiometer (Seca, Hamburg, Germany), with shoes and socks removed and head in the Frankfort plane. Using non-elastic tape (Seca, Hamburg, Germany), waist circumference was measured during mid-expiration at the midpoint between the lower costal margin and the level of the anterior superior iliac crest, and hip circumference was measured around the widest portion of the buttocks. Body weight was assessed to the nearest 0.05 kg using an electric scale (A&D Instruments, Adelaide, Australia). In accordance with the 2007 World Health Organization (WHO) criteria for BMI, a child was classified as overweight/obese if their BMI z score was greater than one SD above the age- and sex-specific mean for the WHO reference sample, which is equivalent to a BMI threshold of 25 kg/m^2^ at age 19 years [28].

### 2.5. Independent Variables: Food Consumption Patterns

Information on food choice was collected using a 28-item food frequency questionnaire (FFQ), which has been validated in this age group and shows acceptable reliability and validity [23]. The 28 items were aggregated into 21 groups, and components/patterns were derived using principle component analysis [23,29]. The number of factors was determined by the minimum eigenvalue principle of a factor analysis of the correlation matrix, with the minimum acceptable eigenvalue >1, the implication being that if an eigenvalue is >1 the derived dimension captures less variability in the data than any single variable. The factors were then subject to orthogonal ‘varimax’ rotation and the factor loadings, the correlation between the derived factors and the underlying variables, were used to interpret (label) each factor. We used a loading of greater than 0.20 to interpret the factor pattern. 

### 2.6. Moderation Variable: Prop Taster Status

Taster status was determined by using taste test strips which contained 3–5 mg of PROP (Sensonics International, NJ, USA) [12]. While PROP does not occur naturally in foods, it elicits a bitter taste stimulus in some individuals, which correlates with taste sensitivity to other bitter substances present in foods [10]. Children were asked to place the taste test strip on the center of their tongue, following the instructions: do not chew or swallow the paper strip, use your index finger to push the strip down entirely on your tongue, while the paper strip is on your tongue count to five slowly in your head, then remove the strip. Subsequently, the children were asked the question: ‘Do you taste anything?’ Children who reported ‘no’, ‘tastes like paper or cardboard’ or ‘tastes good’ were classified as non-tasters. Children who reported that the strip tastes ‘gross’, ‘bad’, ‘bitter’, ‘sour’, ‘yucky’ and ‘spicy’ were all recorded as tasters. Additionally, children who demonstrated classic rejection signs, such as grimacing or frowning, and children who immediately removed the strip because of the reportedly horrific taste were classified as tasters. Children who reported they ‘sort of tasted something’ but the taste was not strong were classified as mild tasters. For analysis, the tasters (*n* = 49) and mild-tasters (*n* = 91) were collapsed in to one group, and herein are referred to as ‘tasters’.effect size; n = number; SD, standard 

### 2.7. Covariates

Statistical models were adjusted for age, sex, ethnicity and SES (see above), as well physical activity levels.

#### Physical Activity

Physical activity levels (mins/wk) were determined using the 47-item youth physical activity questionnaire (YPAQ), which quantifies the frequency, time, and duration of a range of physical and sedentary activities for both week and weekend days over the past 7 days [30].

### 2.8. Statistical Analysis

Statistical analyses were performed using Jamovi (2019, Version 1.0.1) [31]. Only participants who had complete body composition and food consumption pattern data were included in the analyses. The significance level was set a priori for all statistical procedures at α=0.05. The corresponding author (LS) had full access to the data in the study and was responsible for the integrity of the data set and the data analysis. Anonymized data will be shared upon reasonable request.

PROP taster status (taster, non-taster) differences in demographic characteristics were assessed using chi-squared tests for categorical variables and *t*-tests for continuous variables. Associations between body composition and food consumption patterns were examined using a series of linear regression models. Model 1: unadjusted univariate analysis was performed in which each dependent variable (%fat, fat mass, FMI, BMI, W:Ht) was regressed against each independent variable (Processed Foods, Fruits and Vegetables, Breakfast Foods). Model 2: unadjusted multivariate analysis was performed in which each dependent variable was regressed against each of the three independent variables. Model 3: multivariate models were adjusted for age, sex, ethnicity, school decile rating and weekly physical activity (mins). Model 4 the adjusted multivariate models were stratified by PROP taster status (taster, non-taster). Model 5: sex × food consumption pattern interactions terms were specified. All regression models were assessed by examination of the model residuals plotted against their normal scores. For *t*-tests effect sizes were calculated as Cohen’s *d*, and for linear regression effects sizes were calculated by dividing the beta coefficient (β) by the standard deviation of β. For both effect size calculations, <0.20 was considered small, > 0.20 to < 0.50 moderate, and > 0.80 large. Raw data are presented as mean (standard deviation) and regression data are presented as β (95% confidence interval).

## 3. Results

### 3.1. Participants

A representative sample of 392 children were recruited, of which 342 (87%) had complete body composition and questionnaire data (Figure 1). Table 1 presents the demographic characteristics of the participants included in the current analysis. Among the 392 participants, 29% were classified as overweight/obese.

### 3.2. Food Consumption Patterns

The principle component analysis for food group consumption patterns is summarized in Table 2. Using the minimum eigenvalue cut off of one, three dimensions were retained. The table shows the correlation of each variable with the three factors. Based on the variables which loaded on each factor, the factors were labeled: Processed Foods, Fruit and Vegetables, and Breakfast Foods. Collectively, the three factors explained 35% of the variance in the measured variables.

### 3.3. Tasters vs. Non-Tasters

Descriptive data for the children classified as tasters (*n* = 140) and non-tasters (*n* = 202) is provided in Table 1. There were no statistical differences between sub-groups for age (*p* = 0.249), overweight/obesity classification (*p* = 0.540), or any body composition variable (*p* = 0.316 to 0.589). There were also no statistical differences between tasters and non-tasters regarding the frequency of consuming Processed Foods (*p* = 0.487), Fruit and Vegetables (*p* = 0.881) or Breakfast Foods (*p* = 0.266). However, a greater number of non-tasters attended low-SES schools (*p* = 0.004). Further, there was a moderate effect of taster status on physical activity (mins/wk), with the non-tasters accumulating 36 min/wk (95% CI: 7, 66, ES: 0.28) more physical activity.

### 3.4. Associations between Body Composition and Food Types

Models 1–3 are reported in Table 3. In fully adjusted multivariate models (Model 3), there were small but statistically significant positive associations between Processed Foods with %fat (*p* = 0.015), fat mass (*p* = 0.004) and FMI (*p* = 0.016). For Fruits and Vegetables there was a small negative association with W:Ht (*p* = 0.025), but no other body composition variable. For Breakfast Foods, there were small negative associations with all body composition variables (*p* ≤ 0.001 to 0.037).

### 3.5. Associations between Body Composition and Food Types: Stratified by Prop Taster Status

Model 4 stratified the data by PROP status, and the findings are reported in Table 4. Breakfast Foods were not associated with any body composition variable for the tasters. However, for the non-tasters there were small to moderate negative associations between Breakfast Foods and each body composition variable (*p* = 0.003–0.045) except W:Ht (*p* = 0.112). For the tasters, there were small to moderate positive associations between Processed Foods with %fat (*p* = 0.030), fat mass (*p* ≤ 0.001) and FMI (*p* = 0.014).

### 3.6. Sensitivity Analysis: Sex Interaction

Model 5 included sex interaction terms. The findings for the tasters are presented first. For all composition variables, the interactions were non-significant for Sex × Processed Foods (*p* = 0.252 to 0.952) and Sex × Fruits and Vegetables (*p* = 0.266 to 0.984). The Sex × Breakfast Foods interaction was not significant for any body composition variable (*p* = 0.270 to 0.315) except BMI. For BMI, the association between breakfast foods and BMI was stronger for males than females (female to male: ß = 0.388, 95%CI: 0.013, 0.762, *p* = 0.042).

For non-tasters, there were non-significant sex interactions (data not shown) for all body composition variable with respect to Processed Foods (*p* = 0.487 to 0.774) and Fruits and Vegetables (*p* = 0.072 to 0.653). The Sex × Breakfast Foods interaction outcomes are reported in Table 5. For each body composition variable, the negative relationship between Breakfast Foods and body composition was stronger for females compared to males. However, the interactions were not significant for BMI (*p* = 0.156) or W:Ht (*p* = 0.227).

## 4. Discussion

The purpose of the current study was to determine whether PROP taster status moderates the relationship between food consumption patterns and body composition in pre-adolescent children. Across the whole sample, Processed Foods were positively associated with body composition, and Breakfast Foods were negatively associated with body composition. These associations were moderated by PROP taster status. While Breakfast Foods were robustly associated with body composition across the whole population sample, these associations were non-significant for the non-tasters. However, Breakfast Foods were inversely associated with body composition for the tasters, particularly for females. Conversely, Processed Foods positively associated body composition for the non-tasters, but not the tasters.

### 4.1. Limitations and Strengths

While our findings are internally robust, this study had potential limitations. First, this was a cross-sectional study, and longitudinal studies are now warranted to help establish temporality and causality. Second, while we did adjust our models for physical activity levels, the non-tasters accumulated less physical activity per week than the tasters. This may have led to a different energy balance between the groups and statistical adjustment may not have fully accounted for this potential confounding. Third, we adjusted our statistical models by using a school-level proxy of SES; further studies would do well to adjust using a more specific family-level indicator of SES. Last, the population sample is close to New Zealand national averages in terms of overweight/obesity and ethnicity, and therefore we are confident that the current findings can be generalized to New Zealand children. Nonetheless, the sample size was not sufficient to determine whether ethnicity further moderated the relationship between taste status and body composition. Additionally, further study is required to generalize these findings to pre-adolescents in other nations. Major strengths of this study include a relatively large and representative cohort of New Zealand-based pre-adolescents, and the use of multiple measures of body composition.

### 4.2. Associations between Food Patterns and Body Composition

The population sample recruited for the current study was generally representative of New Zealand-based children, including overweight/obesity status (29% vs 32%) [2], sex and ethnicity (New Zealand European: 81% vs. 74%; Māori: 11% vs. 15%) [32]. Across the representative population sample, there were small positive associations between Processed Foods and three body composition variables (%fat, fat mass, FMI), and a small negative association between Breakfast Foods and each of the body composition variables. For Fruits and Vegetables, there was one small association with W:Ht. As such, the most robust associations between body composition with food consumption patterns were with Breakfast Foods followed by Processed Foods, with a likely negligible effect of Fruits and Vegetables. These findings are in-line with previous studies in children reporting a positive association between processed foods and overweight/obesity [33,34]. Additionally, a low intake of breakfast foods, i.e., breakfast skipping, has also been associated with greater prevalence of overweight/obesity [35,36,37]. Conversely, there is considerable evidence from systematic reviews that regularly eating breakfast protects children from becoming overweight/obese [38,39]. By way of rationale, processed foods tend to be high-fat and calorie dense, and children with who prefer or have access to such foods are more likely to have lower general diet quality. Similarly, skipping breakfast can lead to excess hunger and overeating, including more energy-dense food [36]. Whereas eating breakfast may lead to overall lower intake energy, due to the consumption of fiber rich foods and lower intake of energy-dense foods [39], and could increase energy expenditure due to improved energy and vigor [39].

### 4.3. Moderating Effect of PROP (Taster) Status

Contrary to previous reports, the PROP tasters and non-tasters in the current study were not different in terms of over-weight and obesity classification, or for any continuous body composition variable. However, PROP taster status did moderate the relationship between food consumption patterns and body composition. Processed Foods positively associated with body composition for the non-tasters, but not the tasters. Conversely, Breakfast Foods were inversely associated with body composition for the tasters, but not for the non-tasters. The latter finding is in-line with evidence from systematic reviews that regularly eating breakfast protects children from becoming overweight/obese [38,39]. The consumption of breakfast may be particularly important for tasters, who are unlikely to consume healthy foods, including ‘bitter tasting’ vegetables, later in the day. Skipping breakfast can lead to excess hunger and overeating, including the consumption of energy-dense food [36].

With respect to Processed Foods, the current findings are in-line with previous studies in children reporting a positive association between the consumption of processed foods and overweight/obesity [33,34]. Additionally, it has previously been reported that children who are non-tasters consume a greater variety of fat [11,12,13]. It has been hypothesized that non-tasters consume higher-fat foods in the diet to provide additional oral stimulation and sensory input [40]. While several studies have [14,15,41] reported no association between PROP status and intake of high-fat foods, it should be noted that these studies either had a small sample size [15,41] or used food diaries rather than FFQs [14,41].

When comparing tasters vs. non-tasters, it should be acknowledged that while there were no significant differences between groups for sex, age, or ethnicity, the non-tasters did attend lower SES (decile) schools (57% vs. 41%) and accumulated more physical activity per week (180 min vs 143 min). Lower SES areas are generally associated with poorer food environments, which provide children with greater access to unhealthy foods. It has been reported [20] that non-taster children who had greater access to unhealthy foods were at the greatest risk for obesity, which could partially explain the relationship between the consumption of processed foods and body composition among the non-tasters in the current study. With respect to physical activity, along with the fact that overweight/obesity status was non-statistically different between groups, it is plausible that the non-tasters consumed greater quantities of food to ensure energy balance. While our statistical models were adjusted for physical activity, such adjustments may not fully compensate for the confounding influence of greater quantities of food consumption, or the interactions between food consumption, physical activity and metabolism.

The sensitivity analysis investigated potential interactions between sex and food consumption patterns for tasters and non-tasters. This analysis was conducted following previous reports suggesting that compared to tasters, non-taster girls are more likely to consume additional daily servings of fat [13], and have higher BMI z-scores and body fat [21]. For the tasters in the current study, there were no consistent relationships between sex and food consumption patterns. However, for the non-tasters the negative relationships between the consumption of breakfast foods and body composition were stronger for females compared to males. While these findings suggest that the consumption of breakfast may be particularly protective for girls, interpretations should be made in light of the small sample size for this sensitivity analysis. 

### 4.4. Implications

Prior to the current research, there was evidence that PROP status is a determinant of food preference in children, and some studies reported associations between PROP status and overweight/obesity in children. However, not all studies reported an association between PROP status and overweight/obesity. Further, no known studies had examined whether PROP status moderates the relationship between food consumption patterns and body composition in children. Findings from the current study indicate that: (i) Processed Foods are positively associated with body composition in the non-tasters; and (ii) the consumption of Breakfast Foods may protect against overweight/obesity in tasters, particularly among girls. When considering that the current study recruited a relatively large and representative sample of New Zealand children, it is natural to speculate that PROP status might serve as a simple, noninvasive screening method to predict food consumption patterns and their association with the development of overweight/obesity in children. However, it should be considered that taste is one determinant of food choice, with other factors which should be considered, including SES and sex. While the current study statistically adjusted for these covariates, further study is warranted to tease out the importance of SES and sex on the relationship between taste, food preference, and obesity in children.

## 5. Conclusions

Sensitivity to PROP does moderate the relationship between food consumption patterns and body composition in pre-adolescent children. We found that Breakfast Foods were inversely associated with body composition for the tasters, particularly for females. Conversely, Processed Foods positively associated with body composition for the non-tasters, but not the tasters. While PROP status might serve as a simple screening method to predict food consumption patterns and propensity towards the development of overweight/obesity in children, further study is warranted to tease out the importance of SES and sex in the relationship between taste, food preference and obesity.

## Figures and Tables

**Table 1:** Participant characteristics (*n* = 342).

	All		Tasters		Non-Tasters			
Categorical Variables	*n* (%)		*n* (%)		*n* (%)		*P*	
*n*	342	(100)	140	(41)	202	(144)		
Female	169	(49)	73	(52)	96	(48)	0.401	
Ethnicity								
New Zealand European	278	(81)	114	(81)	164	(81)	0.250	
Māori	37	(11)	11	(8)	26	(13)		
Pacific	22	(6)	12	(9)	10	(5)		
Not Recorded	5	(2)	3	(2)	2	(1)		
School Year/Grade								
4	72	(21)	24	(17)	48	(24)	0.521	
5	96	(28)	41	(29)	55	(27)		
6	111	(33)	47	(34)	64	(32)		
7	63	(18)	28	(20)	35	(17)		
School Decile								
Low (≤5)	174	(51)	58	(41)	116	(57)	0.004	
High (>5)	168	(49)	82	(59)	86	(43)		
Obesity status								
Overweight/Obese	99	(29)	38	(27)	61	(30)	0.540	
Non-Overweight	243	(71)	102	(73)	141	(70)		
Continuous variables	Mean (SD)		Mean (SD)		Mean (SD)		*P*	ES
Age (years)	9.56	(1.12)	9.64	(1.05)	9.50	(1.17)	0.248	0.13
Body Fat (%)	19.8	(9.40)	20.2	(9.82)	19.6	(9.12)	0.589	0.06
Fat Mass (Kg)	7.18	(5.08)	7.43	(5.57)	7.01	(4.71)	0.445	0.08
Fat Mass Index (kg/m^2^)	3.68	(2.37)	3.75	(2.53)	3.63	(2.26)	0.653	0.05
Body Mass Index (kg/m^2^)	0.41	(1.18)	0.34	(1.23)	0.45	(1.15)	0.426	−0.09
Waist to Height Ratio (W:Ht)	0.44	(0.05)	0.43	(0.05)	0.44	(0.05)	0.366	−0.10
Physical Activity (mins)	165	(135)	143	(116)	180	(146)	0.015	−0.27
Processed Foods	0.00	(1.24)	0.08	(2.06)	−0.06	(1.69)	0.487	0.08
Fruit/Veg	0.00	(1.56)	−0.02	(1.51)	0.01	(1.60)	0.881	−0.02
Breakfast Foods	0.00	(1.85)	0.09	(1.21)	−0.06	(1.26)	0.266	0.12

Abbreviations: ES, effect size; n = number; SD, standard deviation.

**Table 2:** Food type factor correlations (*n* = 342).

	Factor 1	Factor 2	Factor 3
Food Items/Group	Processed Food	Fruit/Veg	Breakfast Food
Fruits	−0.02	**0.41**	−0.02
Vegetables	−0.01	**0.45**	−0.02
Trim milk	0.01	**0.22**	**−0.45**
Milk	0.11	0.07	**0.54**
Cheese	0.04	**0.27**	**0.32**
Yoghurt	0.19	**0.27**	−0.02
Ice cream	**0.24**	−0.07	−0.06
Processed meat	**0.29**	0.13	−0.06
Other meats	**0.25**	**0.21**	−0.06
Fish	**0.27**	0.04	−0.16
Nondairy drinks	**0.31**	−0.18	−0.05
Breakfast cereals	0.17	0.18	**0.25**
White bread	**0.30**	**−0.19**	0.03
Brown/Wholemeal bread	−0.12	**0.37**	0.09
Rice, rice-based dishes	**0.25**	0.02	**−0.29**
Pasta, noodles	**0.24**	**0.16**	**−0.31**
Salty snacks	**0.25**	−0.13	0.03
Biscuits, cakes, muffins, doughnuts, fruit pies	0.12	**0.15**	**0.25**
Lollies (candies/sweets)	0.24	**−0.19**	**0.16**
Sweet snacks	**0.33**	−0.08	0.13
Spreads	**0.28**	0.11	0.08
			
Eigenvalue	3.5	2.4	1.5
Variance explained (%)	17	11	7
Cumulative Variance (%	17	28	35

Note: Bold numbers represent variables with a factor loading >0.2.

**Table 3:** Linear associations between body composition measures and food types (*n* = 342).

	Univariate					Multivariate					Multivariate (adjusted)				
	*β*	LCI	UCI	*P*	ES	*β*	LCI	UCI	*P*	ES	*β*	LCI	UCI	*P*	ES
Body Fat (%)															
Processed	0.616	0.077	1.150	0.025	0.121	0.785	0.253	1.317	0.004	0.154	0.647	0.128	1.167	0.015	0.130
Fruit/Veg	−0.780	−1.420	−0.144	0.016	−0.130	−0.824	−1.448	−0.200	0.010	−0.137	−0.419	−1.040	0.200	0.184	−0.070
Breakfast	−1.220	−2.020	−0.422	0.003	−0.161	−1.352	−2.143	−0.561	<0 .001	−0.178	−1.440	−2.204	−0.679	<0.001	−0.192
Fat Mass (kg)															
Processed	0.473	0.185	0.762	0.001	0.172	0.561	0.275	0.846	< 0.001	0.204	0.416	0.137	0.695	0.004	0.153
Fruit/Veg	−0.418	−0.761	−0.074	0.017	−0.129	−0.454	−0.789	−0.119	0.008	−0.140	−0.260	−0.594	0.073	0.125	−0.080
Breakfast	−0.581	−1.010	−0.149	0.009	−0.142	−0.679	−1.104	−0.255	0.002	−0.166	−0.729	−1.138	−0.319	<0.001	−0.178
Fat Mass Index (kg/m^2^)															
Processed	0.178	0.042	0.313	0.010	0.139	0.220	0.087	0.354	0.001	0.172	0.163	0.031	0.295	0.016	0.129
Fruit/Veg	−0.238	−0.397	−0.078	0.004	−0.157	−0.251	−0.408	−0.095	0.002	−0.166	−0.146	−0.304	0.011	0.069	−0.096
Breakfast	−0.274	−0.475	−0.072	0.008	−0.143	−0.310	−0.509	−0.112	0.002	−0.162	−0.333	−0.527	−0.140	<0.001	−0.175
Body Mass Index (kg/m^2^)															
Processed	0.039	−0.029	0.107	0.264	0.061	0.054	−0.014	0.122	0.117	0.085	0.018	−0.050	0.085	0.602	0.019
Fruit/Veg	−0.114	−0.194	−0.034	0.005	−0.151	−0.117	−0.197	−0.037	0.004	−0.155	−0.067	−0.147	0.014	0.105	−0.098
Breakfast	−0.088	−0.189	0.014	0.090	−0.092	−0.095	−0.196	0.006	0.065	−0.100	−0.106	−0.205	−0.007	0.037	−0.095
Waist: Height															
Processed	0.001	−0.002	0.004	0.454	0.041	0.002	−0.001	0.005	0.230	0.065	0.000	−0.002	0.003	0.577	0.031
Fruit/Veg	−0.006	−0.009	−0.002	0.001	−0.177	−0.006	−0.009	−0.002	< 0.001	−0.180	−0.004	−0.007	0.000	0.025	−0.124
Breakfast	−0.003	−0.007	0.001	0.137	−0.081	−0.003	−0.008	0.001	0.113	−0.085	−0.004	−0.009	0.000	0.035	−0.114

Adjusted model: age, sex, ethnicity, socioeconomic status, and weekly physical activity (mins). Abbreviations: *β* = beta; LCI = 95% lower confidence interval; UCI = 95% upper confidence interval; ES = effect size (*β/standard deviation).*

**Table 4:** Linear associations between body composition measures and food types stratified by 6-n-propylthiouracil (PROP) taster status (tasters *n* = 140; non-tasters *n* =202).

	Tasters (*n* = 140)					Non-Tasters (*n* = 202)				
	*β*	LCI	UCI	*P*	ES	*β*	LCI	UCI	*P*	ES
Body Fat (%)										
Processed	0.878	0.087	1.669	0.030	0.184	0.317	−0.413	1.048	0.392	0.061
Fruit/Veg	−0.515	−1.626	0.597	0.361	−0.079	−0.362	−1.138	0.413	0.358	−0.065
Breakfast	−0.999	−2.293	0.295	0.129	−0.123	−1.503	−2.488	−0.518	0.003	−0.211
Fat Mass (kg)										
Processed	0.753	0.314	1.193	< 0.001	0.279	0.008	−0.362	0.378	0.966	0.003
Fruit/Veg	−0.221	−0.839	0.397	0.481	−0.060	−0.279	−0.671	0.114	0.163	−0.095
Breakfast	−0.433	−1.152	0.287	0.236	−0.094	−0.783	−1.282	−0.285	0.002	−0.209
Fat Mass Index (kg/m^2^)										
Processed	0.258	0.052	0.464	0.014	0.211	0.042	−0.139	0.223	0.647	0.032
Fruit/Veg	−0.135	−0.424	0.154	0.357	−0.080	−0.149	−0.341	0.043	0.126	−0.107
Breakfast	−0.206	−0.542	0.131	0.228	−0.099	−0.361	−0.604	−0.117	0.004	−0.203
Body Mass Index (kg/m^2^)										
Processed	0.069	−0.035	0.173	0.193	0.116	−0.035	−0.130	0.060	0.473	−0.052
Fruit/Veg	−0.063	−0.209	0.084	0.399	−0.077	−0.068	−0.169	0.033	0.187	−0.095
Breakfast	−0.034	−0.204	0.136	0.692	−0.034	−0.131	−0.259	−0.003	0.045	−0.144
Waist: Height										
Processed	0.002	−0.003	0.006	0.411	0.075	−0.000	−0.004	0.004	0.941	−0.005
Fruit/Veg	−0.003	−0.009	0.003	0.333	−0.090	−0.004	−0.008	0.000	0.056	−0.139
Breakfast	−0.003	−0.010	0.004	0.426	−0.070	−0.004	−0.010	0.001	0.112	−0.115

All models adjusted for: age, sex, ethnicity, socioeconomic status, and weekly physical activity (mins). Abbreviations: *β* = beta; LCI = 95% lower confidence interval; UCI = 95% upper confidence interval; ES = effect size (*β/standard deviation).*

**Table 5:** Linear associations between body composition measures and breakfast foods in PROP non-tasters: sex interaction effects.

		Female (*n* = 106) - Male (*n* = 96)			
	*β*	LCI	UCI	P	ES
Body Fat (%)	−2.233	−4.222	−0.243	0.028	−0.124
Fat Mass (kg)	−1.105	−2.119	−0.098	0.032	−0.376
Fat Mass Index (kg/m^2^)	−0.553	−1.045	−0.062	0.028	−0.395
Body Mass Index (kg/m^2^)	−0.188	−0.448	0.073	0.156	−0.263
Waist: Height	−0.007	−0.017	0.004	0.227	−0.080

All models adjusted for: age, sex, ethnicity, socioeconomic status, and weekly physical activity (mins). Abbreviations: *β* = beta; LCI = 95% lower confidence interval; UCI = 95% upper confidence interval; ES = effect size (*β/standard deviation).*

## Supplementary Materials

The following are available online at https://www.mdpi.com/2072-6643/11/9/2037/s1, study questionnaire.
