# Supplementary material for: Food Consumption Patterns and Body Composition in Children: Moderating Effects of Prop Taster Status
Source: Nutrients. 2019 Aug 31;11(9):2037. doi: 10.3390/nu11092037 (PMC6770641; doi:10.3390/nu11092037)
Supplement: Supplementary file 1 [file nutrients-11-02037-s001.pdf]

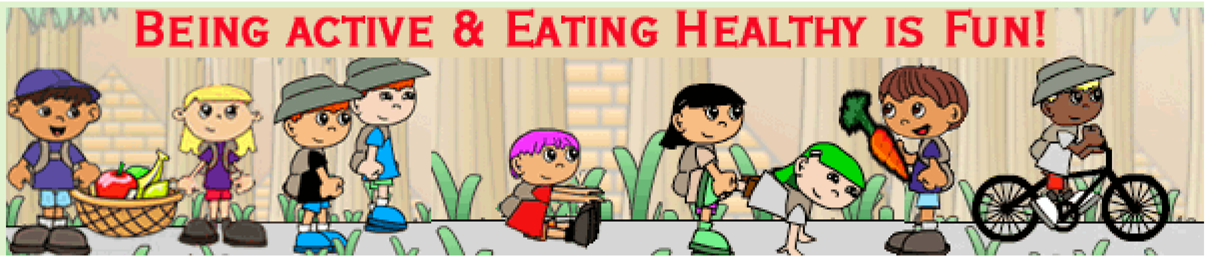

This questionnaire asks about the things that you eat and drink, how well you sleep, and how much and what kinds of physical activity you do.

This questionnaire must be filled out by the participant (child) and a parent/guardian - <u>together as a team unless noted.</u>

- Please answer the questions honestly and as accurately as you can.
- This is not a test – there are no right or wrong answers to the questions.
- We will not tell anyone your answers.
- The questions about your parent/guardian are for whom you live with or who takes care of you.

Study ID Number: ______________

Demographic:

- PARENTS please answer all the questions as honestly and accurately as you can.
- Please do not leave any lines blank, so if you are a single parent put N/A in parent 2 questions.

1. Participant's (child) name: _______________________________

2. Date of birth of child (dd/mm/yy): __________/__________/__________

3. Child sex:
   - ☐ Male
   - ☐ Female

4. Ethnicity:
   - ☐ New Zealand European
   - ☐ Māori
   - ☐ Samoan
   - ☐ Cook Island Māori
   - ☐ Tongan
   - ☐ Chinese
   - ☐ Indian
   - ☐ Other ___________________________

5. Parent/Guardian marital status:
   - ☐ Single, not living with a partner
   - ☐ Single, living with a partner
   - ☐ Married
   - ☐ Divorced
   - ☐ Never Married

6. Parent/Guardian (parent 1), occupation/job:____

7. Parent/ Guardian (parent 1), please tick all school/academic qualifications:
   - ☐ None
   - ☐ NCEA Level 1 /5th Form
   - ☐ NCEA Level 2 /6th Form
   - ☐ NCEA Level 3 /7th Form
   - ☐ Degree
   - ☐ Honours Degree
   - ☐ Post-Graduate Diploma
   - ☐ Master's Degree
   - ☐ Doctorate

8. Parent/Guardian (parent 2), occupation/job: ____

9. Parent/ Guardian (parent 2), please tick all academic qualifications:
   - ☐ None
   - ☐ NCEA Level 1 /5th Form
   - ☐ NCEA Level 2 /6th Form
   - ☐ NCEA Level 3 /7th Form
   - ☐ Honours Degree
   - ☐ Post-Graduate Diploma
   - ☐ Master's Degree
   - ☐ Doctorate

10. Household income:
   - ☐ $0
   - ☐ $1 - $5,000
   - ☐ $5001 - $10,000
   - ☐ $10,001 - $15,000
   - ☐ $15,001 - $20,000
   - ☐ $20,001 - $25,000
   - ☐ $25,001 - $30,000
   - ☐ $30,001 - $35,000
   - ☐ $35,001 - $40,000
   - ☐ $40,001 - $50,000
   - ☐ $50,001 - $60,000
   - ☐ $60,001 - $70,000
   - ☐ $70,000 - $100,000
   - ☐ $100,001 - $150,000
   - ☐ $150,001 - Above

11. Child home street address: ____

12. Child home flat/house number:____

13. Child home postal code: ____

14. School name: ____

Physical Activity – Part 1

- The following questions are about the activities the participant (child) usually does.
- Parent AND Participant please fill this section out together.
- Please answer all questions as honestly and accurately as you can.
- Please **tick a box on every line** in the questionnaire.

AVAILABILITY

1. Do you consider your school walking distance from your home?

   ☐ Yes?

   ☐ No?

2. Are there playgrounds or parks within walking distance from your home where you can play?

   ☐ Yes?

   ☐ No?

How many times did you do visit playgrounds or parks close to your home in the PAST 7 DAYS?

	Each day that you did this, how long did you normally do it for?	How many days did you do this activity?			
		Never	Once	2 to 3 times	4 or more times
How often do you visit playgrounds or parks close to your home	hrs min	☐	☐	☐	☐

How many times did you do the following PHYSICAL activities in the PAST 7 DAYS?

3. **ACTIVITIES AT SCHOOL IN THE PAST 7 DAYS**

	Each day that you did this, how long did you normally do it for?	How many days did you do this activity?			
		Never	Once	2 to 3 times	4 or more times
PE class	hrs min	☐	☐	☐	☐
Walk to school	hrs min	☐	☐	☐	☐
Cycle to school	hrs min	☐	☐	☐	☐
Travel to school by car / bus	hrs min	☐	☐	☐	☐
Travel to school by skateboard/scooter/ bike		☐	☐	☐	☐

4. **ACTIVITIES OUTSIDE SCHOOL IN THE PAST 7 DAYS**

	Each day that you did this, how long did you normally do it for?	How many days did you do this activity?			
		Never	Once	2 to 3 times	4 or more times
SPORTS ACTIVITIES (NOT AT SCHOOL)					
Aerobics	hrs min	☐	☐	☐	☐
Softball / Tee ball	hrs min	☐	☐	☐	☐
Basketball / Volleyball	hrs min	☐	☐	☐	☐
Cricket	hrs min	☐	☐	☐	☐
Dancing	hrs min	☐	☐	☐	☐
Football	hrs min	☐	☐	☐	☐
Gymnastics	hrs min	☐	☐	☐	☐
Hockey (field or ice)	hrs min	☐	☐	☐	☐
Martial arts (e.g. karate or judo)	hrs min	☐	☐	☐	☐
Netball	hrs min	☐	☐	☐	☐

	Each day that you did this, how long did you normally do it for?	How many days did you do this activity?			
		Never	Once	2 to 3 times	4 or more times
Rugby/League	hrs min	☐	☐	☐	☐
Running or jogging	hrs min	☐	☐	☐	☐
Swimming lessons	hrs min	☐	☐	☐	☐
Swimming for fun	hrs min	☐	☐	☐	☐
Tennis/badminton/squash/ other racquet sport	hrs min	☐	☐	☐	☐
LEISURE TIME ACTIVITIES					
Bike riding (not to or from school)	hrs min	☐	☐	☐	☐
Trampolining	hrs min	☐	☐	☐	☐
Bowling	hrs min	☐	☐	☐	☐
Household or farm chores	hrs min	☐	☐	☐	☐
Playing on playground equipment	hrs min	☐	☐	☐	☐
Playing with pets or horse riding	hrs min	☐	☐	☐	☐
Rollerblading / roller-skating	hrs min	☐	☐	☐	☐
Playing on scooter	hrs min	☐	☐	☐	☐
Skateboarding	hrs min	☐	☐	☐	☐
Skiing, snowboarding, or sledging	hrs min	☐	☐	☐	☐
Skipping	hrs min	☐	☐	☐	☐
Walking the dog	hrs min	☐	☐	☐	☐
Walking for exercise	hrs min	☐	☐	☐	☐

	Each day that you did this, how long did you normally do it for?	How many days did you do this activity?			
		Never	Once	2 to 3 times	4 or more times
Art & craft (e.g. pottery, sewing, drawing, painting)	hrs min	☐	☐	☐	☐
Doing homework	hrs min	☐	☐	☐	☐
Listening to music	hrs min	☐	☐	☐	☐
Playing indoors with toys	hrs min	☐	☐	☐	☐
Playing board games / cards	hrs min	☐	☐	☐	☐
Playing musical instrument	hrs min	☐	☐	☐	☐
Reading	hrs min	☐	☐	☐	☐
Sitting talking	hrs min	☐	☐	☐	☐
Talking on the phone	hrs min	☐	☐	☐	☐
Any other activities you do (Please write it in here)	hrs min	☐	☐	☐	☐

5. **ACTIVITIES OUTSIDE SCHOOL ON A SCHOOL DAY IN THE PAST 7 DAYS**

	On the SCHOOL DAYS that you did this, how long did you normally do it for?	On how many SCHOOL DAYS did you do this activity?			
		Never	One day	2 to 3 days	4 or more times
Playing computer games (like Xbox, Play station / game boy/ DS/ PSP)	hrs min	☐	☐	☐	☐
Using computer / internet	hrs min	☐	☐	☐	☐
Watching TV / DVDs	hrs min	☐	☐	☐	☐

6. **ACTIVITIES OUTSIDE SCHOOL ON A WEEKEND DAY IN THE PAST 7 DAYS**

	On the WEEKEND DAYS that you did this, how long did you normally do it for?	On how many WEEKEND DAYS did you do this activity?		
		Never	One day	Two days
Playing computer games (like Xbox, play station / game boy/ DS/ PSP)	hrs min	☐	☐	☐
Using computer / internet	hrs min	☐	☐	☐
Watching TV / DVDs	hrs min	☐	☐	☐

Physical Activity – Part 2

- The following questions are about the activities the participant (child) usually does.
- Parent AND Participant please fill this section out together.
- Put a check by the number that best describes your response to the question.
- Answers are on a point system from 1-4.
- 1 being the lowest given score.
- 4 being the highest given score.

E.g. I like to eat ice cream more than anything else.

1	2	3	✓ 4
Always NO	Sometimes NO	Sometimes YES	Always YES

1. I like playing outdoor games and sports.

1	2	3	4

2. I like getting sweaty when I exercise or play hard.

1	2	3	4

3. I have more fun playing games and sports than anything else.

1	2	3	4

4. I like to exercise lots.

1	2	3	4

5. I am told that I am good at games and sports.

1	2	3	4

6. I feel really tired after I play games and sports.

1	2	3	4

7. I get nervous and worried about playing games and sports.

1	2	3	4

8. I get teased by other kids when I play games and sports.

1	2	3	4

9. I think that the more exercise you get the better.

1	2	3	4

10. I make a lot of friends when I play games and sports.

1	2	3	4

11. I enjoy exercise a lot.

**1**	**2**	**3**	**4**

12. I try to stay in good **shape** (explained as having a good looking body).

**1**	**2**	**3**	**4**

13. I wish I could play more games and sports.

**1**	**2**	**3**	**4**

14. I think that I will feel really good after I play hard.

**1**	**2**	**3**	**4**

15. I do not mind getting out of breath after I play hard.

**1**	**2**	**3**	**4**

16. I think it is very important to always be in good shape.

**1**	**2**	**3**	**4**

17. Playing games and sports is my favourite thing.

**1**	**2**	**3**	**4**

18. I really like to run a lot.

**1**	**2**	**3**	**4**

19. I think exercise is very important for my **health** (Explain as well being & strong).

**1**	**2**	**3**	**4**

20. I look forward to playing sports and games.

**1**	**2**	**3**	**4**

21. I like to burn lots of energy by playing hard.

**1**	**2**	**3**	**4**

22. I think that exercise is the most important thing for good health.

**1**	**2**	**3**	**4**

23. I really like to exercise.

**1**	**2**	**3**	**4**

24. I feel good when I run hard.

**1**	**2**	**3**	**4**

25. I am popular when I play games and sports.

**1**	**2**	**3**	**4**

Nutrition:

- We would like to know about your general eating habits.
- Please answer all questions as honestly and accurately as you can.
- Please **tick a box for every question** on the questionnaire.
- **Questions 1-11 are for the CHILDREN to answer with parent's assistance if needed.**
- **Questions 12-37 are for the PARENT/GUARDIAN to answer.**

1. Do you like the taste of onions?

   ☐ Yes?

   ☐ No?

2. Do you like the taste of broccoli?

   ☐ Yes

   ☐ No

3. How often do you usually have these meals (more than a glass of milk or fruit juice) during the WEEK?
   Please choose only one of the following per meal:

	Breakfast	Lunch	Dinner
Never	☐	☐	☐
One day	☐	☐	☐
Two days	☐	☐	☐
Three days	☐	☐	☐
Four days	☐	☐	☐
Five days	☐	☐	☐

4. How often do you usually have these meals (more than a glass of milk or fruit juice) during the WEEKEND?
   Please choose only one of the following per meal:

	Breakfast	Lunch	Dinner
Never	☐	☐	☐
One day	☐	☐	☐
Both days	☐	☐	☐

5. On school days during lunch break do you generally…
Please tick one from the following:

Eat food bought at school or ordered through the school (E.g. Subway, Pita Pit, Sushi etc.)	☐
Eat a packed lunch brought from home	☐
Go home for lunch	☐
Eat food bought on the way to school	☐
Don't eat lunch	☐

6. For each of these questions, please tick which answer you think is right.

	None	Hardly any	Some	Quite a lot	Lots
Do you think that you eat a lot of fruit?	☐	☐	☐	☐	☐
Do you think that you eat a lot of vegetables?	☐	☐	☐	☐	☐

7. For each of these questions, please tick which answer you think is right

	Never	Not often	Sometimes	Quite often	Always
If you ask for fruit that you like will your parents buy it for you?	☐	☐	☐	☐	☐
If you ask for vegetables that you like will your parents buy it for you?	☐	☐	☐	☐	☐
Are there usually different kinds of fruit available at home?	☐	☐	☐	☐	☐
Are there usually different kinds of vegetables available at home?	☐	☐	☐	☐	☐
Is there usually fruit at home that you like?	☐	☐	☐	☐	☐
Are there usually vegetables at home that you like?	☐	☐	☐	☐	☐

8. In the past week, at home…………*Tick one box per line*

	Yes	No
**a.** were there fruit or vegetables on the kitchen counter or somewhere in the open	☐	☐
**b.** was there fruit juice, fruit or cut up vegetables in the fridge as a snack?	☐	☐

9. How much do you like each of these foods? *Tick one box per question.*

	Like	They're ok	Dislike		Like	They're ok	Dislike
**a.** Apples				**m.** Butter			
**b.** Oranges				**n.** Pasta			
**c.** Bananas				**o.** Rice			
**d.** Strawberries				**p.** Cake			
**e.** Grapes				**q.** Pizza			
**f.** Pears				**r.** Sausages			
**g.** Peas				**s.** Chicken			
**h.** Carrots				**t.** Potatoes*			
**i.** Broccoli				**u.** Fish			
**j.** Salad				**v.** Milk			
**k.** Tomatoes				**w.** Ice Cream			
**l.** Sweetcorn				**x.** Chocolate			

* Not hot chips/potato chips

10. How healthy do you think each of these foods are? *Tick one box per question*

	Healthy	They're ok	Not healthy		Healthy	They're ok	Not Healthy
**a.** Apples				**m.** Butter			
**b.** Oranges				**n.** Pasta			
**c.** Bananas				**o.** Rice			
**d.** Strawberries				**p.** Cake			
**e.** Grapes				**q.** Pizza			
**f.** Pears				**r.** Sausages			
**g.** Peas				**s.** Chicken			
**h.** Carrots				**t.** Potatoes*			
**i.** Broccoli				**u.** Fish			
**j.** Salad				**v.** Milk			
**k.** Tomatoes				**w.** Ice Cream			
**l.** Sweetcorn				**x.** Chocolate			

* Not hot chips/potato chips

11. How many times a week do you usually eat or drink…? *(Please tick ONE box for each item)*

		Never	Less than once a week	Once a week	2 - 4 days a week	5 - 6 days a week	Every day, once a day	Every day, more than once
**1**	Fruits							
**2**	Vegetables (excluding potato)							
**3**	Trim milk (green) [including on cereals, milo, hot chocolate]							
**4**	Milk (blue) [including on cereals, milo, hot chocolate]							
**5**	Cheese							
**6**	Yoghurt							
**7**	Ice-cream							
**8**	Processed meat (such as meat pies, sausage, sausage roll, salami, luncheon, bacon, ham)							
**9**	Other meats (such as mince, beef, chicken)							
**10**	Fish (including canned tuna or salmon, fish cakes, fish fingers, fish pie, battered fish)							
**11**	Fruit juice (such as Orange juice, Apple juice, Raro, Refresh, Keri, Twist, Ribena)							
**12**	Diet fizzy drinks (such as Diet Coke, Pepsi Max, Sprite Zero and any other "light" or "sugar free" varieties.							
**13**	Fizzy drinks (such as Coke, Pepsi, Sprite, L&P, Fanta, Ginger Beer)							
**14**	Breakfast cereals							
**15**	White bread							
**16**	Brown /Wholemeal bread							
**17**	Rice, rice based dishes							
**18**	Pasta (such as spaghetti, macaroni), noodles							
**19**	Potato (such as mashed, boiled)							
**20**	Potato chips, potato snacks, corn chips							
**21**	Hot chips, wedges, French fries							
**22**	Biscuits, cakes, muffins, doughnuts, fruit pies							
**23**	Snack bars (such as muesli bar, fruit bar, rice bubble bar)							
**24**	Lollies							
**25**	Chocolate, Chocolate bars							
**26**	Tomato sauce, Ketchup							
**27**	Peanut butter, Nutella							
**28**	Jam, Honey							

Questions 12-37 are for the PARENT/GUARDIAN to answer.

12. How often do you usually have these meals (more than a drink) DURING THE WEEK?

Please choose only one of the following:

	Breakfast	Lunch	Dinner
Never	☐	☐	☐
One day	☐	☐	☐
Two days	☐	☐	☐
Three days	☐	☐	☐
Four days	☐	☐	☐
Five days	☐	☐	☐

13. How often do you usually have these meals (more than a drink) DURING THE WEEKEND?

Please choose only one of the following:

	Breakfast	Lunch	Dinner
Never	☐	☐	☐
One day	☐	☐	☐
Both days	☐	☐	☐

14. On work days during lunch break do you generally:

Please choose only one of the following:

- ☐ Eat food bought at work
- ☐ Eat a lunch brought from home
- ☐ Go home for lunch
- ☐ Eat food bought on the way to work
- ☐ Don't eat lunch

15. When you eat takeaways, do you usually pick healthier options if these are available?

Please choose only one of the following:

- ☐ Yes
- ☐ No
- ☐ I don't eat takeaways

16. How often do you eat takeaways (such as McDonalds, KFC, Fish 'n' chips, Domino's Pizza, Hell Pizza, Pizza Hut, Country Fried Chicken, and Asian Takeaways)? *Please choose only one of the following:*

	Alone or with friends/colleagues	With family
Never	☐	☐
Less than once a week	☐	☐
Once a week	☐	☐
2-4 days a week	☐	☐
5-6 days a week	☐	☐
Once a day	☐	☐
More than once a day	☐	☐

Section 9: Dietary Habits

This section is about your usual eating patterns. When answering these questions please think back over the past 4 weeks. Remember to think about all meals (that is breakfast, lunch and dinner) as well as snacks and times when you eat both at home and away from home.

17. On average, how many slices of bread/toast OR bread rolls do you eat per day?

- ☐ None, I don't eat bread or toast
- ☐ Less than one per day
- ☐ 1-2 per day
- ☐ 3-4 per day
- ☐ 5-6 per day
- ☐ 7 or more per day
- ☐ Don't know

18. What type of bread, rolls or toast do you eat most of?

- ☐ White
- ☐ High fibre white
- ☐ Light grain bread (e.g. Molenburg, Freya's, Ploughmans, And MacKenzie High Country)
- ☐ Heavy grain bread (e.g. Vogels and Burgen)
- ☐ Other
- ☐ Don't know

19. In the past four weeks, which of the following have you eaten at all?

- ☐ Red meat- such as beef, pork, mutton, lamb and goat
- ☐ Chicken- such as chicken breast, drumsticks, or whole chickens, but not chicken nuggets or chicken roll
- ☐ Processed meats- such as ham, bacon, sausages, chicken roll, luncheon, canned corned beef, pastrami, and salami
- ☐ Seafood- such as fish or shellfish
- ☐ None
- ☐ Don't know

20. How often do you EAT:

	Red meat	Chicken
Never		
Less than once per week		
1-2 times per week		
3-4 times per week		
5-6 times per week		
7 or more times per week		
Don't know		

21. How often do you REMOVE:

	Excess fat from meat	Skin from chicken
Never		
Rarely		
Sometimes		
Regularly		
Always		
Don't know		

22. How often do you eat processed meat products? Processed meat includes ham, bacon, sausages, luncheon, canned corned beef, pastrami, and salami.

- ☐ Never
- ☐ Less than once per week
- ☐ 1-2 times per week
- ☐ 3-4 times per week
- ☐ 5-6 times per week
- ☐ 7 or more times per week
- ☐ Don't know

23. How often do you eat fresh or frozen fish or shellfish? Do not include battered/fried or canned fish or shellfish.

- [ ] Never
- [ ] Less than once per week
- [ ] 1-2 times per week
- [ ] 3-4 times per week
- [ ] 5-6 times per week
- [ ] 7 or more times per week
- [ ] Don't know

24. How often do you eat battered or fried fish or shellfish? This may include battered or deep fried fish bought from the 'Fish and Chip' shop.

- [ ] Never
- [ ] Less than once per week
- [ ] 1-2 times per week
- [ ] 3-4 times per week
- [ ] 5-6 times per week
- [ ] 7 or more times per week
- [ ] Don't know

25. How often do you eat canned fish or shellfish? Canned fish includes products such as tuna, salmon, and sardines.

- [ ] Never
- [ ] Less than once per week
- [ ] 1-2 times per week
- [ ] 3-4 times per week
- [ ] 5-6 times per week
- [ ] 7 or more times per week
- [ ] Don't know

26. On average, how many servings of fruit- fresh, frozen, canned or stewed- do you eat per day?
A serving is the same as a medium piece of fruit such as an apple, or two small pieces of fruit such as two apricots, or half a cup of stewed fruit. *Do not include fruit juice or dried fruit.*

- [ ] Never, I don't eat fruit
- [ ] Less than one serving per day
- [ ] 1 serving

- [ ] 2 servings
- [ ] 3 servings
- [ ] 4 or more servings
- [ ] Don't know

27. On average, how many servings of vegetables- fresh, frozen or canned- do you eat per day?
A serving is the same as one potato/kumara, half a cup of peas, or a cup of salad. For example, 2 potatoes + ½ cup of peas = 3 servings. *Do not include vegetable juices.*

- [ ] Never, I don't eat vegetables
- [ ] Less than one serving per day
- [ ] 1 serving
- [ ] 2 servings
- [ ] 3 servings
- [ ] 4 or more servings
- [ ] Don't know

28. What type of milk do you use the most of?

- [ ] None, I don't use milk
- [ ] Whole or standard milk (dark blue or silver)
- [ ] Reduced fat (light blue)
- [ ] Skim or trim (green or yellow)
- [ ] Soy milk
- [ ] Other (such as rice, goats milk)
- [ ] Don't know

29. What type of butter or margarine spread do you use the most of?

- [ ] Never, I don't use butter or margarine as spread
- [ ] Butter (including semi-soft)
- [ ] Butter and margarine blend
- [ ] Margarine- Full fat (e.g. Canola, Sunflower, and Olive oil based)
- [ ] Light or reduced fat margarine (e.g. Canola, Sunflower, and Olive oil based)
- [ ] Plant sterol margarine- full and low fat varieties (e.g. Proactive or Logicol)
- [ ] Don't know

30. What type of fat or oil do you use most often when cooking?

- [ ] None, I don't use fat or oil
- [ ] Butter
- [ ] Margarine
- [ ] Butter blend
- [ ] Oil
- [ ] Dripping or Lard
- [ ] Other
- [ ] Don't know

31. How often do you add salt to your food after it has been cooked or prepared?

- [ ] Never
- [ ] Rarely
- [ ] Sometimes
- [ ] Regularly
- [ ] Always
- [ ] Don't know

32. How often do you choose low or reduced fat varieties of foods instead of the standard variety?

- [ ] Never
- [ ] Rarely
- [ ] Sometimes
- [ ] Regularly
- [ ] Always
- [ ] Don't know

33. How often do you choose low or reduced salt varieties of foods instead of the standard variety?

- [ ] Never
- [ ] Rarely
- [ ] Sometimes
- [ ] Regularly
- [ ] Always
- [ ] Don't know

34. How often do you eat hot chips, French fries, wedges, or kumara chips? Think about lunch, dinner, and snacks.

- [ ] Never
- [ ] Less than once per week
- [ ] 1-2 times per week
- [ ] 3-4 times per week
- [ ] 5-6 times per week
- [ ] 7 or more times per week
- [ ] Don't know

35. How often do you drink fruit juices and drinks? *Do not include diet or diabetic varieties.* Fruit juices and drinks include freshly squeezed varieties, and brands such as Just Juice, Fresh-up, Keri, Golden Circle, Ribena, Thextons, McCoy and Charlie's. **Excludes**- 'diet varieties', soft drinks and energy drinks, flavoured waters (e.g. H2Go), and sports waters (e.g. Charlies Sports Water, Mizone, and Aqua-shot).

- [ ] Never
- [ ] Less than once per week
- [ ] 1-2 times per week
- [ ] 3-4 times per week
- [ ] 5-6 times per week
- [ ] 7 or more times per week
- [ ] Don't know

36. How often do you drink soft drinks or energy drinks? *Do not include diet varieties.* Soft drinks are often carbonated or 'fizzy' and include Coca-Cola, Pepsi, Lemonade, Ginger Beer, Energy drinks (e.g. 'V', Red Bull, and Lift Plus), PowerAde, E2, and G-force.

- [ ] Never
- [ ] Less than once per week
- [ ] 1-2 times per week
- [ ] 3-4 times per week
- [ ] 5-6 times per week
- [ ] 7 or more times per week
- [ ] Don't know

37. How often do you eat lollies, sweets, chocolate, and confectionary?

- [ ] Never
- [ ] Less than once per week
- [ ] 1-2 times per week
- [ ] 3-4 times per week
- [ ] 5-6 times per week
- [ ] 7 or more times per week
- [ ] Don't know

Sleep:

- The following questions are to be answered by the PARENT about their child's sleep habits in the past week. If last week was unusual for a specific reason, choose the most recent typical week.
- Please **tick a box for every question** on the questionnaire.
- Always if something occurs every night.
- Usually if it occurs 5 to 6 times a week.
- Sometimes if it occurs 2 to 4 times a week.
- Rarely if it occurs once a week.
- Never if it occurs less than once a week.

BEDTIME:

Write in your child's usual bedtime: School night _______ - _______ pm

*School night = has school the next day

*Non-school night = has no school the next day Non-school night _______ - _______ pm

	7 Always	5-6 Usually	2-4 Sometimes	1 Rarely	0 Never
1. Child goes to bed at the same time at night	( )	( )	( )	( )	( )
2. Child falls asleep within 20 minutes after going to bed	( )	( )	( )	( )	( )
3. Child falls asleep alone in own bed	( )	( )	( )	( )	( )
4. Child falls asleep in parent's or sibling's bed	( )	( )	( )	( )	( )
5. Child needs parent in the room to fall asleep	( )	( )	( )	( )	( )
6. Child struggles at bedtime (cries, refuses to stay in bed, etc.)	( )	( )	( )	( )	( )
7. Child is afraid of sleeping in the dark	( )	( )	( )	( )	( )
8. Child is afraid to sleep alone	( )	( )	( )	( )	( )

SLEEP BEHAVIOR:

Write in your child's usual amount of sleep each day. (Combining night time sleep and naps)

*School day = has school the next day ____________hours and ___________ minutes

*Non-school day = has no school the next day ____________hours and ___________ minutes

	7 Always	5-6 Usually	2-4 Sometimes	1 Rarely	0 Never
9. Child sleeps too little	( )	( )	( )	( )	( )
10. Child sleeps the right amount	( )	( )	( )	( )	( )
11. Child sleeps about the same amount each day	( )	( )	( )	( )	( )
12. Child wets the bed at night	( )	( )	( )	( )	( )
13. Child talks during sleep	( )	( )	( )	( )	( )
14. Child is restless and moves a lot during sleep	( )	( )	( )	( )	( )
15. Child sleepwalks during the night	( )	( )	( )	( )	( )
16. Child moves to someone else's bed during the night (parent, brother, sister, etc.)	( )	( )	( )	( )	( )
17. Child grinds teeth during sleep (your dentist may have told you this)	( )	( )	( )	( )	( )
18. Child snores loudly	( )	( )	( )	( )	( )
19. Child seems to stop breathing during sleep	( )	( )	( )	( )	( )
20. Child snorts and/or gasps during sleep	( )	( )	( )	( )	( )
21. Child has trouble sleeping away from home (visiting relatives, and holidays)	( )	( )	( )	( )	( )
22. Child awakens during the night screaming, sweating, and inconsolable	( )	( )	( )	( )	( )
23. Child awakens alarmed by a frightening dream	( )	( )	( )	( )	( )

WAKING DURING THE NIGHT:

	7 Always	5-6 Usually	2-4 Sometimes	1 Rarely	0 Never
24. Child awakes once during the night	( )	( )	( )	( )	( )
25. Child awakes more than once during the night	( )	( )	( )	( )	( )

Write the number of minutes a night waking usually lasts:

___________hours and __________ minutes

MORNING WAKE UP

Write in the time your child usually wakes up in the morning:

*School day = has school that day — School day _______ - _______ am

*Non-school day = has no school that day — Non-school day _______ - _______ am

	7 Always	5-6 Usually	2-4 Sometimes	1 Rarely	0 Never
26. Child wakes up by him/herself	( )	( )	( )	( )	( )
27. Child wakes up in a negative mood	( )	( )	( )	( )	( )
28. Adults or siblings wake up child	( )	( )	( )	( )	( )
29. Child has difficulty getting out of bed in the morning	( )	( )	( )	( )	( )
30. Child takes a long time to become alert in the morning	( )	( )	( )	( )	( )

Daytime Sleepiness:

	7 Always	5-6 Usually	2-4 Sometimes	1 Rarely	0 Never
31. Child seems tired	( )	( )	( )	( )	( )

During the past week, your child has appeared very sleepy or fallen asleep during the following (Tick all that apply):

	1 Not Sleepy	2 Very Sleepy	3 Falls Asleep
32. Watching TV	( )	( )	( )
33. Riding in car	( )	( )	( )

Birth History (Parents/Guardians)

- PARENTS, please answer these questions as best as you can.
- The following questions are in regards to the birth of your child.

1. Was your son/daughter a twin?

   ☐ Yes?

   ☐ No?

2. Do you know the exact weight of your son/ daughter at birth?

   ☐ Yes?

   ☐ No?

3. If yes to Q2, please state your child's weight: __________Kg

4. If no to Q2 was your child:

   ☐ Less than 2.5 kg

   ☐ More than 4 kg

5. Do you know the exact gestational week (e.g., 37 weeks is normal) that you delivered your son/ daughter?

6. If yes to Q5 please state the gestational age: __________weeks

7. If no to Q5, did your deliver you son/ daughter:

   ☐ In less than 37 weeks

   ☐ More than 37 weeks
